# SARS in Three Categories of Hospital Workers, Hong Kong

**DOI:** 10.3201/eid1008.040041

**Published:** 2004-08

**Authors:** Joseph T.F. Lau, Xilin Yang, Ping-Chung Leung, Louis Chan, Eliza Wong, Carmen Fong, Hi-Yi Tsui

**Affiliations:** * The Chinese University of Hong Kong, Hong Kong, China

**Keywords:** SARS, incidence study, Hong Kong, Chinese, hospital infection, nosocomial infection, healthcare workers, research

## Abstract

We analyzed attack rates for severe acute respiratory syndrome (SARS) in three categories of hospital workers (nurses, nonmedical support staff, and other technical or medical staff) in all public hospitals in Hong Kong that had admitted SARS patients. Of 16 such hospitals, 14 had cases. The overall attack rate was 1.20%. Nonmedical support staff had the highest attack rate (2.73%). The odds ratios of group nonmedical support staff versus those of nurses and of nonmedical support staff versus other technical or medical staff were 2.30 (p < 0.001) and 9.78 (p < 0.001), respectively. The number of affected staff and attack rates were significantly correlated with the number of SARS patients admitted (r = 0.914 and 0.686, respectively). Affected patients were concentrated in three hospitals and in the earlier phase of the epidemic. Cleaning and clerical staff on hospital wards were at a much higher risk.

## Abstract 2

The SARS attack rate for hospital workers in Hong Kong was 1.20% and was significantly higher in nonmedical support staff.

The global epidemic of severe acute respiratory syndrome (SARS) occurred in Hong Kong, mainland China, and other countries from March to June in 2003. The cases in Hong Kong and mainland China accounted for 84.1% of all cases worldwide (1,755 and 5,327, respectively); the number of deaths accounted for 70.9% of all SARS-related deaths worldwide (298 and 348, respectively) (1). The first major outbreak in Hong Kong occurred in the Prince of Wales Hospital around March 10, 2003. It resulted in 138 SARS patients, 69% of whom were hospital workers (2). In Hong Kong, 360 hospital workers contracted SARS, a figure that represented 20.5% of all case-patients on the island (3). A study reported that 40 hospital workers in a community hospital in Hong Kong were affected during a 6-week period (March 25-May 5, 2003); the attack rates were 6.1, 10.2, 8.8, 2.0, 0.0, and 0.0 per 1,000, respectively, in these 6 weeks (4).

In Canada, the first large SARS outbreak also occurred in a community hospital, affecting 128 patients (36.7% of all hospital staff). The attack rates among nurses ranged from 10.3% to 60.0%, depending on which department they were serving (5). In mainland China, nosocomial infections played an important role in the SARS outbreak, especially in the first phase of the epidemic (6-8). Nosocomial infection was the most important cause of the SARS outbreak in the Haidian district, Beijing (7). Hospital workers were therefore at high risk of contracting SARS. Improved hospital infection control likely contributed substantially to the control of the SARS epidemic in Hong Kong (9).

A case-control study of 72 hospital workers with SARS and 144 matched controls found that inconsistent use of goggles, gowns, gloves, and caps (unadjusted odds ratio [OR] = 2.42-20.54, p < 0.05), as well as perceived inadequate training and perceived inadequate supply of protective equipment were significantly associated with higher risk for nosocomial infection (10). Another study in China showed that good ventilation, isolation of SARS patients, and use of personal protection equipment were key means of preventing healthcare workers from becoming infected (11).

Because a substantial number of hospital workers contracted SARS in Hong Kong and in other places, documenting the attack rate in different hospitals in Hong Kong was warranted. Such information would reflect the degree of exposure to relevant occupational hazards among different types of hospital workers in Hong Kong.

This study gives an account of the attack rates of workers in all public hospitals in Hong Kong that had admitted SARS patients. The attack rates of three categories of hospital workers, as well as relevant attack rates in the earlier and later phases of the epidemic, were compared. The attack rates were also correlated with the number of SARS patients admitted to the individual hospitals.

## Methods

For all hospitals that had admitted SARS patients in Hong Kong, the numbers of probable SARS patients and of hospital staff in three job categories were obtained. These three categories included nurses (group N), nonmedical support staff (group S; healthcare assistants, ward assistants [cleaning staff], general service assistant [clerical staff]), and other medical or technical staff (group O; physicians, allied health workers, technicians, pharmacists, dieticians, radiologists, radiographers, and medical students, and the like). All were full-time staff. The Hospital Authority and individual hospitals kept lists of infected workers who were hospital staff members. These lists were provided to the authors, with data already grouped into the three categories and the two time periods; no further breakdown of the data was available. Most data were obtained from the Hospital Authority; supplementary data were obtained from a few hospitals. The number of these three types of workers who became probable SARS patients, according to the World Health Organization definition (1), was recorded. These figures were further stratified into two groups: patients whose onset of symptoms occurred 1) before April 17, or 2) on or after April 17, which was approximately the mid-point of the epidemic. (The first patient was admitted on March 4, 2003, and the onset of the last case was on May 31, 2003). Attack rates for the three categories of hospital workers were obtained by dividing the relevant number of hospital care workers contracting SARS by the total number of relevant staff members.

Chi-square test and Fisher exact test were used to test the significance of differences in proportions. Spearman correlation analysis was performed to examine the association between the number of SARS patients admitted into a hospital and the number of healthcare workers who contracted SARS in the same hospital. Analysis of variance (ANOVA) and Kruskal-Wallis test were used to compare differences in attack rates among the three types of workers. SPSS for Windows Release 11.0.1 (SPSS Inc., Chicago, IL) was used for the data analysis; p < 0.05 was considered to be significant. Differences in attack rates among the 16 hospitals were tested by using Fisher-Freeman-Halton test (StatXact-4 version 4.0.1, Cytel Software Corporation, Cambridge, MA).

## Results

### Infected Staff and Attack Rates

A total of 1,755 SARS patients were reported in Hong Kong; they were hospitalized in 16 of the 27 hospitals governed by the Hospital Authority. Fourteen of these 16 hospitals had at least one hospital staff member who contracted SARS. In other words, 2 of the 16 hospitals (hospital 2 and hospital 4, which admitted 7 and 17 SARS patients, respectively) had a zero attack rate (Table 1).

**Table 1:** Number of hospital workers, SARS-affected hospital workers, and SARS patients admitted to hospitals and attack rates

Hospital no.^a^	No. of SARS patients admitted to hospitals	No. of hospital staff who contracted SARS				Attack rates (%)			
		Group N	Group S	Group O	Overall^a^	Group N	Group S	Group O	Overall^b^
1	3	0	0	2	2	0.00	0.00	0.73	0.18
2	7	0	0	0	0	0.00	0.00	0.00	0.00
3	17	6	2	0	8	1.19	0.96	0.00	0.79
4	17	0	0	0	0	0.00	0.00	0.00	0.00
5	24	1	3	0	4	0.33	1.29	0.00	0.64
6	29	7	3	1	11	0.95	0.83	0.23	0.71
7	36	2	0	0	2	0.12	0.00	0.00	0.07
8	53	1	2	1	4	0.26	1.80	0.41	0.54
9	82	5	5	1	11	0.42	1.11	0.18	0.50
10	83	8	6	0	14	0.51	1.15	0.00	0.48
11	85	6	6	1	13	0.47	1.41	0.14	0.54
12	114	18	14	4	36	3.58	6.93	1.37	3.61
13	128	15	7	0	22	0.87	1.42	0.00	0.68
14	188	13	14	2	29	1.01	3.36	0.26	1.17
15	326	64	54	2	120	4.66	13.30	0.21	4.38
16	563	35	18	10	63	2.76	3.92	1.53	2.64
Pooled	1,755	181	134	24	339	1.21	2.73	0.29	1.20
p value						< 0.001^c^	< 0.001^c^	< 0.001^d^	< 0.001^c^
Spearman correlation coefficients		0.883***	0.928***	0.525*	0.914*	0.737*	0.865***	0.390	0.686**

^a^All hospital workers, including all three groups (group N, nurses; group S, nonmedical support staff; and group O, other technical and medical staff). ^b^All hospitals that had at least admitted one SARS patient. ^c^p values derived from Pearson Chi-square test and comparing the attack rates among all the 16 hospitals. ^d^p values derived from Fisher-Freeman-Halton test and comparing the attack rates among all the 16 hospitals: *, p < 0.05; **, p < 0.01; and ***, p < 0.001.

The total number of affected hospital workers in these 16 hospitals was 339 (i.e., 94.2% of all 360 affected hospital workers in Hong Kong). The other 21 (5.8%) affected hospital staff worked in six other hospitals that had not admitted SARS patients. The distribution of the 339 cases is analyzed in this article.

The number of affected staff in 2 hospitals (hospitals 15 and 16) accounted for 54.0% of the 339 cases in the 16 Health Authority hospitals (Table 1). The number of affected staff in an individual hospital ranged from 0 to 120 (median = 11, interquartile range = 24.8) (Table 1). The overall attack rates for all three types of hospital staff was 1.20% for the 16 hospitals. These rates ranged from 0% to 4.38%, a significant variation (p < 0.001, Table 1). The overall mean and median of the 16 hospital attack rates for all workers were 1.06% and 0.59%, respectively (Table 2). The overall attack rates for all workers were >2% in three hospitals (hospitals 12, 15, and 16; Table 1). When these three hospitals were removed from the analysis, the overall attack rate was 0.54% and the mean and median of the 13 hospital attack rates were 0.48% and 0.54%, respectively (data not shown in table).

**Table 2:** Mean and median attack rates of the 16 hospitals by job categories

	Mean of attack rates (%)	SD	Median of attack rates (%)	Interquartertile range	Range
Group N	1.07	1.38	0.49	0.99	0.00-4.66
Group S	2.34	3.43	1.22	2.76	0.00-13.30
Group O	0.32	0.49	0.16	0.37	0.00-1.53
Overall^a^	1.06	1.31	0.59	0.82	0.00-4.38
p value	0.035^b^		0.015^c^		

^a^Overall: all the hospital workers, including all three groups (group N, nurses; group S, nonmedical support staff; group O, other technical and medical staff). ^b^p values for testing differences among group N, S, and O (ANOVA). ^c^p values for testing differences among group N, S, and O (Kruskal-Wallis test).

### Attack Rates by Category of Hospital Worker

Attack rates in the three job category groups (group N, S, and O) of hospital workers are listed in Table 1. The ranges of attack rates for the three groups were 0%-4.66% (group N), 0.0%-13.3% (group S), and 0.0%-1.53% (group O). The pooled attack rates for these three groups were 1.21%, 2.73%, and 0.29%, respectively, in the 16 hospitals (Table 1). ORs for comparing the S and N, O and N, and S and O groups were 2.30 (p < 0.001), 0.24 (p < 0.001), and 9.78 (p < 0.001), respectively. The differences in both the mean and median attack rates for the three categories were also significant (p = 0.035, ANOVA test, and p = 0.015 and p = 0.015, Kruskal-Wallis test) (Table 2).

### Associations between Numbers of SARS Patients Admitted and Hospital Attack Rates

The number of affected staff was strongly correlated with the number of admitted SARS patients for all the three groups: group N (Spearman r = 0.883, p < 0.001), group S (Spearman r = 0.928, p < 0.001), and group O (Spearman r = 0.525, p <0.05) (Table 1). Similar significant associations between attack rates and number of admitted SARS patients were observed for groups N and S but not for group O (Spearman r = 0.737, 0.865, and 0.39, respectively) (Table 1).

### Comparison of Attack Rates in First Two Phases of Epidemic

The overall attack rates for all hospital workers in the first phase of the epidemic (before April 17, 2003: 0.98%) were higher than those for the second phrase (on or after April 17, 2003: 0.22%) (Table 3). This finding was true for all three groups of workers (group N: 0.99% vs. 0.22%; group S: 2.24% vs. 0.50%; group O: 0.21% vs. 0.07%). When data from individual hospitals were examined, however, the trend was not always consistent.

**Table 3:** Attack rate among hospitals by job categories and time period

Hospital no.	Group N		Group S		Group O		Overall^a^	
	Onset before 7/4 (%)	Onset after 7/4 (%)	Onset before 7/4 (%)	Onset after 7/4 (%)	Onset before 7/4 (%)	Onset after 7/4 (%)	Onset before 7/4 (%)	Onset after 7/4 (%)
1	0.00	0.00	0.00	0.00	0.36	0.36	0.09	0.09
2	0.00	0.00	0.00	0.00	0.00	0.00	0.00	0.00
3	0.40	0.80	0.00	0.96	0.00	0.00	0.20	0.60
4	0.00	0.00	0.00	0.00	0.00	0.00	0.00	0.00
5	0.00	0.33	0.43	0.86	0.00	0.00	0.16	0.48
6	0.00	0.95	0.28	0.55	0.23	0.00	0.13	0.58
7	0.00	0.12	0.00	0.00	0.00	0.00	0.00	0.07
8	0.26	0.00	0.90	0.91	0.41	0.00	0.40	0.14
9	0.42	0.00	1.11	0.00	0.18	0.00	0.50	0.00
10	0.26	0.26	0.19	0.96	0.00	0.00	0.17	0.31
11	0.47	0.00	1.41	0.00	0.14	0.00	0.54	0.00
12	3.38	0.21	6.44	0.53	1.02	0.34	3.31	0.31
13	0.87	0.00	1.42	0.00	0.00	0.00	0.68	0.00
14	1.01	0.00	2.88	0.49	0.13	0.13	1.05	0.12
15	4.08	0.61	11.82	1.68	0.21	0.00	3.87	0.53
16	2.36	0.40	3.27	0.68	1.07	0.46	2.18	0.47
Pooled (16 hospitals)	0.99	0.22	2.24	0.50	0.21	0.07	0.98	0.22
Ratio of pooled rates (phase 1 vs. 2)	4.50:1		4.48:1		3.00:1		4.45:1	
p values (among 16 hospitals)	< 0.001^b^	< 0.001^c^	< 0.001^c^	0.036^c^	0.002^c^	0.048^c^	< 0.001^b^	< 0.001^c^

^a^Overall: all the hospital workers, including all three groups (N, S, and O). ^b^Pearson Chi-square test. ^c^Fisher-Freeman-Halton test.

## Discussion

The overall attack rate for all workers in the 16 hospitals was 1.2%. Staff members working in 14 hospitals contracted SARS, although the attack rates varied significantly among hospitals. The attack rate was >2% in three hospitals, which had 219 (60.8%) of the total 360 cases in Hong Kong. Compared to the overall attack rate (1.2%), the attack rate was much lower (0.54%) for the other 13 hospitals; the pooled overall attack rate for the 16 hospitals was also much lower (0.47%) in the second phase of the epidemic. In other words, nosocomial infection of hospital workers in Hong Kong was concentrated in three hospitals and in the earliest phase of the epidemic (the overall attack rates in the earlier phase were 2.18%, 3.37%, and 3.81% for these three hospitals).

Attack rates were also associated with the number of SARS patients admitted into the individual hospitals. Five of the 16 hospitals admitted >100 patients. In terms of number of affected workers and attack rates, these five hospitals were also the top five of the 16 studied hospitals (except for hospital 13, which ranked seventh in terms of attack rates). Theoretically, viral load, inadequate manpower, inadequate equipment, and inadequate time for training were possible explanations for the observed associations. If the SARS epidemic resurges on a sizable scale, some consideration should be given to the number of patients to be admitted to a hospital. Yet, without further data, the exact reasons were not clear.

The attack rates also differed significantly among the three studied occupational groups. Support staff, such as healthcare assistants, cleaners, and clerical staff working on the wards (group S), had much higher attack rates, when compared to nurses (group N) and other categories of hospital workers, including physicians (group O). The attack rate of nonmedical support staff (group S) was higher than those of the other two groups in 10 of the 16 studied hospitals. Nonmedical support staff accounted for 134 (39.5%) of the 339 SARS patients among hospital workers, despite the fact that only approximately 17.3% of all Hospital Authority staff belonged to this group. Furthermore, 12 of the 16 hospitals had cases in nonmedical support staff (attack rate 0.83%-13.3% [mean 2.34%]). Even when the later phase of the epidemic was considered, the attack rates of nonmedical support staff were still relatively high (Table 3). Three of the six Hospital Authority staff who died of SARS also belonged to this group. In short, nonmedically trained support staff were exposed to a disproportionately high risk for nosocomial transmission of SARS. Apparently, infection control training was also offered to this group. However, the exact amount of training or assessment of how well the training was understood by this group was not documented.

Extra protection is required to protect this staff group in the infection control campaign in Hong Kong. Insufficient emphasis may have been given to address the special needs of this group during the first SARS epidemic in Hong Kong, as well as during the post-SARS period. Infection control training and policies may need to be tailored for different occupational groups.

Nonmedical support staff, in general, were not contacting SARS patients as frequently as nurses did. This finding suggests that the fomite theory and the aerosol theory of transmission could not be dismissed. Another study investigating nosocomial infection in Hong Kong (10) reported that breakthrough transmission was likely responsible for nosocomial infection of hospital workers. Inconsistent use of personal protection equipment, perceived inadequate supply of such equipment, inadequate training, and perceived lack of knowledge about infection control were all statistically significant predictors of such breakthrough transmissions.

Nonmedical support staff may have been more likely to be lacking infection control knowledge, either as a result of receiving inadequate training or being unable to benefit fully from it. Whether they were adequately trained to use their personal protective equipment correctly is not clear. For instance, some preliminary studies mentioned that some workers were wearing or taking off such equipment in the wrong sequence (Lau et al., unpub. data). In Hong Kong, many support staff were likely to be middle-aged persons, who had a relatively low level of education (many of them did not finish secondary schools). Tailored infection control training and surveillance programs are hence warranted to ensure that this group of workers is sufficiently protected from the occupational hazard of contracting SARS. Practice exercises may also be an effective preventive measure.

These findings do not mean that nurses were not under a high level of occupational hazard. More than 50% of the affected workers were nurses. In six hospitals, nurses' attack rates were close to or higher than 1%; the maximum was 4.66%. The correlations between number of SARS patients admitted and the number of affected workers were much stronger in the nonmedical support and nursing groups, when compared with that of other medical staff). This finding is understandable as most members of these two groups were working on the frontlines.

Most affected staff (94.2%) were working in hospitals that had been taking care of SARS patients. Further transmission through social contacts had not been a major factor of transmission among hospital workers (10). Nosocomial infection was therefore likely to be responsible for most transmission. Hospital workers in Hong Kong were well supported and appreciated by Hong Kong citizens and the mass media for their professionalism in treating SARS patients. Whether such strong media interest in their experiences and commitment influenced hospital workers in their decision to serve in high-risk environments, at times even when protection might not have been adequate, is of interest. Questions such as how conformity and peer pressure affected the decisions of individual workers who felt that they had to work under suboptimal infection control conditions are worth exploring.

The pooled attack rates (for 16 hospitals) were 0.22, 0.50, and 0.07, respectively, for nurses, nonmedical support, and other medical and technical staff when only cases of later onset (on or after April 17, 2003) were considered. When cases with earlier onset (before April 17, 2003) were considered, the rates were higher: 0.99, 2.24, and 0.21, respectively. The respective ratios of the two phases of the epidemic were 4.50:1, 4.48:1, and 3.00:1 for the three types of hospital workers and 4.45:1 for hospital workers overall (Table 3). This may be due to a reduction in the number of patients admitted after April 17 (approximately 18.3% of all cases; the number of patients admitted in the two phases was hence 3.92:1) or to improvement of infection control measures. The overall attack rate ratio (4.45:1, 0.98%/0.22%) was very similar to the overall admission rate for the two phases (4.46:1), although the two ratios were not conceptually equivalent. It, however, gives a clue that the decrease in exposure may have played a relatively important role in the decreased attack rate in the second phase. Improved infection control in the second phase may not be the primary reason for the decrease in the attack rate over time. If proper training, supply of personal protective equipment, infection control procedures, nonexcessive number of patients per hospital, and other measures are ensured, nosocomial infection of hospital workers should be avoidable. On the other hand, hospital workers, especially nonmedical support staff, should be aware that they are facing a certain level of occupational risk.

The study has some limitations. First, only macro-level data were used. Since no individual data were available, factors associated with nosocomial infection could not be studied. Similarly, no clinical data were reported. Some hospital workers may have been infected in the community. However, another case-control study showed that social contact with other infected colleagues was not a significant factor associated with likelihood of infection among hospital workers (10). The chance of nosocomial infection was therefore very high for these staff who contracted SARS. Another study suggested that asymptomatic transmission among hospital workers was not prevalent (5). Second, different types of workers were included in the three studied job categories, and some heterogeneity across these different types of workers with the categories may exist. The available data do not permit further breakdown. The classification of affected workers into the three categories was arbitrary and may also have affected results. Further, no additional data exist to compare the conditions on infection control and other measures taken within the two analyzed phases of the epidemic, making definite interpretation impossible. The study, however, documents one of the most important scenarios of nosocomial infection among hospital workers. The results should help us to learn from this very costly lesson.

This study was funded by the Chinese University of Hong Kong.
